# Exogenous GABA enhances muskmelon tolerance to salinity-alkalinity stress by regulating redox balance and chlorophyll biosynthesis

**DOI:** 10.1186/s12870-019-1660-y

**Published:** 2019-02-01

**Authors:** Xiaoqing Jin, Tao Liu, Jiaojiao Xu, Zixing Gao, Xiaohui Hu

**Affiliations:** 1 College of Horticulture, Northwest A & F University, Yangling, 712100 Shaanxi China; 2 Key Laboratory of Protected Horticultural Engineering in Northwest, Ministry of Agriculture, Yangling, 712100 Shaanxi China

**Keywords:** Salinity-alkalinity stress, γ-Aminobutyrate (GABA), Muskmelon, Hydrogen peroxide (H_2_O_2_), Ascorbate-glutathione (AsA-GSH) cycle, Chlorophyll biosynthesis

## Abstract

**Background:**

Salinity-alkalinity stress is one of the major abiotic stresses affecting plant growth and development. γ-Aminobutyrate (GABA) is a non-protein amino acid that functions in stress tolerance. However, the interactions between cellular redox signaling and chlorophyll (Chl) metabolism involved in GABA-induced salinity-alkalinity stress tolerance in plants remains largely unknown. Here, we investigated the role of GABA in perceiving and regulating chlorophyll biosynthesis and oxidative stress induced by salinity-alkalinity stress in muskmelon leaves. We also evaluated the effects of hydrogen peroxide (H_2_O_2_), glutathione (GSH), and ascorbate (AsA) on GABA-induced salinity-alkalinity stress tolerance.

**Results:**

Salinity-alkalinity stress increased malondialdehyde (MDA) content, relative electrical conductivity (REC), and the activities of superoxide dismutase (SOD), ascorbate peroxidase (APX) and dehydroascorbate reductase (DHAR). Salinity-alkalinity stress decreased shoot dry and fresh weight and leaf area, reduced glutathione and ascorbate (GSH and AsA) contents, activities of glutathione reductase (GR) and monodehydroascorbate reductase (MDAR). By contrast, pretreatment with GABA, H_2_O_2_, GSH, or AsA significantly inhibited these salinity-alkalinity stress-induced effects. The ability of GABA to relieve salinity-alkalinity stress was significantly reduced when the production of endogenous H_2_O_2_ was inhibited, but was not affected by inhibiting endogenous AsA and GSH production. Exogenous GABA induced *respiratory burst oxidase homologue* D (*RBOHD*) genes expression and H_2_O_2_ accumulation under normal conditions but reduced the H_2_O_2_ content under salinity-alkalinity stress. Salinity-alkalinity stress increased the accumulation of the chlorophyll synthesis precursors glutamate (Glu), δ-aminolevulinic acid (ALA), porphobilinogen (PBG), uroporphyrinogen III (URO III), Mg-protoporphyrin IX (Mg-proto IX), protoporphyrin IX (Proto IX), protochlorophyll (Pchl), thereby increasing the Chl content. Under salinity-alkalinity stress, exogenous GABA increased ALA content, but reduced the contents of Glu, PBG, URO III, Mg-proto IX, Proto IX, Pchl, and Chl. However, salinity-alkalinity stress or GABA treated plant genes expression involved in Chl synthesis had no consistent trends with Chl precursor contents.

**Conclusions:**

Exogenous GABA elevated H_2_O_2_ may act as a signal molecule, while AsA and GSH function as antioxidants, in GABA-induced salinity-alkalinity tolerance. These factors maintain membrane integrity which was essential for the ordered chlorophyll biosynthesis. Pretreatment with exogenous GABA mitigated salinity-alkalinity stress caused excessive accumulation of Chl and its precursors, to avoid photooxidation injury.

**Electronic supplementary material:**

The online version of this article (10.1186/s12870-019-1660-y) contains supplementary material, which is available to authorized users.

## Background

Muskmelon (*Cucumis melon* L.) is an important horticultural fruit that is widely cultivated in northern China [1]. This region is undergoing soil salinization and alkalization [2]. Muskmelons are sensitive to salt conditions, and the melon industry is negatively affected by soil salinization and alkalization. Soil salinization and alkalization can occur via natural and man-made processes; it has become one of the main adverse environmental stresses on crop plants [1, 3]. Plants perceive and defend against environmental stresses via a range of biochemical reaction mechanisms and gene expression networks [3], redox balance [4], and complex signal transduction pathways [5]. Reactive oxygen species (ROS) accumulate in response to salinity-alkalinity stress and trigger changes in cellular antioxidant capacity, ultimately leading to oxidative damage [3]. The antioxidant defense system has a key role in balancing the ROS levels in plants [6, 7]. Generally, the superoxide (O_2_^−^) is converted into hydrogen peroxide (H_2_O_2_) through superoxide dismutase (SOD). Subsequently, the H_2_O_2_ is converted into H_2_O and oxygen which is mainly regulated by catalase (CAT), ascorbate-glutathione (AsA-GSH) cycle, and other antioxidase and antioxidants [8]. AsA and GSH are major nonenzymatic antioxidants, while ascorbate peroxidase (APX), monodehydroascorbate reductase (MDAR), dehydroascorbate reductase (DHAR), and glutathione reductase (GR) have essential roles in the AsA-GSH cycle [8, 9]. The AsA-GSH cycle may have an important role in maintaining the cell redox status in plants, especially under abiotic stress [10].

Chlorophyll (Chl) is the main pigment facilitating photosynthesis; it absorbs and captures solar energy and mediates energy transduction. However, excessive Chl accumulation can cause photooxidation injury and lead to leaf senescence [11, 12]. The Chl biosynthesis pathway has many steps, and any abnormality in one step will affect Chl synthesis [3]. Our previous study reported that salinity-alkalinity stress destroyed the structure and function of photosystem II in muskmelon [1]. A study of tomato seedlings reported that salinity-alkalinity stress disrupted Chl metabolism by preventing the conversion of uroporphyrinogen III (URO III) to protoporphyrin IX (Proto IX), which reduced the Chl content [3]. In addition, the abiotic stress caused cell redox state imbalance, which may disturb the coordinated chlorophyll synthesis [13]. So, how to regulate cell redox homeostasis to maintain normal Chl synthesis is crucial for plant growth and development under salinity-alkalinity stress.

Extensive research has been devoted to enhancing the complex abiotic stress tolerance of crops through breeding programs. Recent studies reported that the application of exogenous factors such as melatonin, polyamines, and γ-aminobutyric acid (GABA) is a simple and effective method to improve plant tolerance and crop yield under salt stress [3, 14, 15]. GABA is a natural non-protein amino acid in animals, plants, and bacteria [13]. In plants, GABA functions as a metabolite or signaling molecule in a number of physiological processes under stress conditions [16–18]. For example, exogenous application of GABA relieved chilling injury of tomato seedlings by regulating antioxidant enzyme activities and subsequent eliminating ROS [19]. Exogenous GABA alleviated the hypoxia damage by accelerating PA biosynthesis and conversion as well as preventing PA degradation in melon plants [20]. Drought induced GABA accumulation increased plant stress response and prevented the water loss [21].

Except for oxidative damage, ROS is also an important signal molecule involved in regulating plant physiology and growth [22–24]. Hu et al. [25] showed that H_2_O_2_ was produced at a specific cellular site and regulated antioxidant enzyme activities. Liu et al. [24] showed that H_2_O_2_ mediated ALA-induced cold resistance. However, few studies have investigated potential interactions between H_2_O_2_, cellular redox signaling, and plant resistance to oxidative stress under GABA-induced salinity-alkalinity stress tolerance, or the relationship between chlorophyll synthesis and GABA under salinity-alkalinity conditions. In the present study, we investigated the relationships among H_2_O_2_, AsA-GSH cycle, and chlorophyll synthesis in GABA-pretreated and untreated leaves of muskmelon plants grown under salinity-alkalinity stress conditions.

## Methods

### Plant materials, growth conditions, and experimental design

Hydroponic experiments were performed at the Northwest A & F University using salt-sensitive muskmelon (*Cucumis melo L.* cv. Yipintianxia No. 208, which were obtained from Shaanxi Qianpu Agricultural Development Co., Ltd., China.) as experimental material [26]. Muskmelon seedlings were cultivated according to the methods of Zhen et al. [18]. Seedlings were transplanted into hydroponic tanks after they developed two true leaves. Experimental treatments started when the plants had four true leaves. A preparation of alkalic salt (NaCl/Na_2_SO_4_/NaHCO_3_/Na_2_CO_3_ 1:9:9:1 M ratio) was added to the hydroponic nutrient solution for a final concentration of 50 mM salt (pH 8.6). Muskmelon leaves were sprayed with 50 mM GABA (Sigma Aldrich, St. Louis, MO, USA) before subjecting plants to normal hydroponic solution (Control) or salinity-alkalinity treatment for 8 h. This GABA concentration was chosen based on previous results [27]. Four general conditions were used in the experiments: (1) H_2_O foliar prespraying under normal nutrient solution cultivation conditions, (Control); (2) 50 mM GABA foliar prespraying under nutrient solution cultivation conditions, (CG); (3) normal nutrient solution containing 50 mM salinity-alkalinity and H_2_O foliar prespraying, (S); (4) 50 mM GABA foliar prespraying under salinity-alkalinity stress, (SG). GABA or H_2_O were sprayed on the leaves to uniformly cover the adaxial and abaxial surfaces, for 8 h before normal hydroponic solution or salinity-alkalinity treatment. All seedlings were grown at the temperature of 28/18 °C (day/night) in a greenhouse, with neutral light and a photoperiod of 14/10 h (day/night).

An inhibitor of endogenous GABA biosynthesis was used to investigate the effects of GABA on plant tolerance to salinity-alkalinity stress. Before S treatment or SG treatment, the seedlings were pretreated with 0.1 mM 3-mercaptopropionic (3-MP) for 12 h. Then, leaves were sprayed with 50 mM GABA, and after 8 h, plants were exposed to salinity-alkalinity stress. Plants were treated for 3 days before measuring plant fresh weight, dry weight, leaf area, malondialdehyde (MDA) content, and relative electrical conductivity (REC).

To investigate the effect of exogenous GABA on antioxidant capacity of muskmelon seedlings under salinity-alkalinity stress, antioxidant enzyme activities and antioxidant contents were measured in plant samples derived from the four treatments (Control, CG, S, and SG) after 3 d of treatment. In the two treatments (Control and CG), H_2_O_2_ contents were measured at 0, 1, 3, 6, 12, 24, 48 and 72 h; *respiratory burst oxidase homologue D* (*RBOHD*) genes expression were measured at 0, 1, 3, 6, 12, 24 h, respectively.

To determine the effects of H_2_O_2_, GSH, and AsA in GABA-induced tolerance response to oxidative stress under salinity-alkalinity stress, muskmelon leaves were pretreated with 5 mM dimethylthiourea (DMTU, scavenges of H_2_O_2_ and superoxide O_2_^−^), 100 μM diphenyleneiodonium (DPI, inhibits oxidative burst and NADPH oxidases that generate H_2_O_2_), 1 mM buthionine sulfoximine (BSO, inhibits GSH biosynthesis), or 1 mM acriflavine (AF, inhibits AsA biosynthesis) for 8 h. Then, the leaves were sprayed with 50 mM GABA, 5 mM H_2_O_2_, 5 mM GSH, or 1 mM AsA. After 8 h, the plants were exposed to salinity-alkalinity stress. After 3 d, the MDA content and Fv/Fm were measured.

To investigate the effects of GABA on Chl biosynthesis, plants were subjected to four different treatments (Control, CG, S, and SG) as described above, and exposed to 3 d of stress. Then, we measured the contents of total Chl, Chl a, Chl b, and Chl precursor including δ-aminolevulinic acid (ALA), porphobilinogen (PBG), uroporphyrinogen III (URO III), Mg-protoporphyrin IX (Mg-proto IX), protoporphyrin IX (Proto IX), and protochlorophyll (Pchl), along with the relative gene expression. GABA and ALA are synthesized from glutamate (Glu), so we also determined the contents of endogenous GABA and Glu after 24, 48, 72 h of stress treatment.

In the same single experiment, three biological replicates were analyzed for each treatment, five seedlings in each replicate were used to perform all the determinations. Growth indices, REC, and contents of Chl and its precursor were assessed with fresh samples. And frozen samples stored at − 80 °C were used for the measurement of MDA content, H_2_O_2_ levels, antioxidant enzyme activities, the contents of glutathione, ascorbate, GABA and Glu, and genes expression. All leaves were washed with distilled water before sampling.

### Measurement of plant growth indices

After 3 d of salinity-alkalinity treatment, plants were washed with distilled water, and then dried off with absorbent paper. Then, the fresh weights of shoot and root were measured. The dry weights of shoot and root were measured after drying the samples for 15 min at 105 °C and for 72 h at 75 °C. All the fresh leaves of each muskmelon seedling (excluding cotyledons) were scanned (Epson Expression 1680 1.0 scanner, Seiko Epson Corp., Tokyo, Japan), and then leaf area was calculated using Image J software, and the total leaf area was expressed as the sum of all leaf areas of a seedling.

### Measurement of F_v_/F_m_, malondialdehyde content, and relative electrical conductivity

Chlorophyll fluorescence (F_v_/F_m_) was measured according to the methods of Pérez-Bueno et al. [28] using the Open FluorCam FC 800-O multispectral fluorescence imager, and analyzed using FluorCam7 software (Photon Systems Instruments, Brno, Czech Republic). MDA contents were measured according to the method of Wu et al. [29]. REC was measured according to method of Zhou and Leul [30].

### Measurement of H_2_O_2_ content

Hydrogen peroxide (H_2_O_2_) was determined by fluorimetry as described by Romero-Puertas et al. [31]. Leaf tissues were extracted in 25 mM H_2_SO_4_ (1:2 *w*/*v*). H_2_O_2_ content was analyzed using homovanillic acid (excitation at 315 nm, emission at 425 nm) and horseradish peroxidase in 50 mM Hepes, pH 7.5 [31]. The H_2_O_2_ concentration was obtained using a standard curve of commercial H_2_O_2_.

### Measurements of antioxidant enzyme activities

Protein contents were measured using the Bradford method [32] with bovine serum albumin as the standard. SOD (EC 1.15.1.1) activity was measured with the SOD activity unit defined as the amount of enzyme needed to inhibit 50% of nitro blue tetrazolium decline per minute at 560 nm absorbance [33]. CAT (EC 1.11.1.6) activity was measured by monitoring the decrease of H_2_O_2_ within 120 s at 240 nm. DHAR (EC 1.8.5.1) activity was measured by dynamically monitoring changes in ascorbate concentration within 180 s at 265 nm. GR (EC 1.6.4.2) activity was assayed by dynamically monitoring decreases in NADPH concentration within 180 s at 340 nm. APX (EC 1.11.1.11) activity was determined by dynamically monitoring decreases in ascorbate concentration within 120 s at 290 nm. MDAR (EC 1.6.5.4) activity was measured by dynamically monitoring decreases in NADPH concentration for 180 s at 340 nm. All of the reactions and measurements were performed according to the methods of Noctor et al. [34].

### Determination of the AsA, DHA, GSH, and GSSG contents

Reduced glutathione (GSH), oxidized glutathione (GSSG) and ascorbate (AsA), and dehydroascorbic acid (DHA) contents were measured according to the methods of Noctor et al. [34].

### Determination of chlorophyll and chlorophyll precursor contents

Chl a, Chl b, and total Chl contents were measured using the method of Goodwin [35]. Proto IX, Mg-proto IX, and Pchl contents were measured according to the method of Hodgins and Van [36]; sample absorption was measured at 575, 590, and 628 nm, respectively. URO III and PBG contents were determined according to the method of Bogorad [37]; sample absorption was measured at 405.5 and 535 nm, respectively. The ALA content was measured according to the method of Morton [38] at 553 nm.

### Expression of genes

The expression of genes was measured by performing real-time quantitative PCR. Total RNA was extracted using Plant RNA Extraction kit (OmegaBio-Tek, Doraville, GA, USA) according to the manufacturer’s protocol. RNA was reverse transcribed using the PrimeScript TM RT Reagent kit with a gDNA Eraser (Takara, Shiga, Japan) according to the manufacturer’s protocol. We measured the relatively expression of *respiratory burst oxidase homologue D* (*RBOHD*) gene and key genes involved in the chlorophyll biosynthetic pathway including aminolevulinic dehydratase (*ALAD*)*,* porphobilinogen deaminase (*PBGD*)*,* Mg-protoporphyrin IX methyltransferase (*CHLM*)*,* protochlorophyllide oxidoreductase (*POR*)*,* chlorophyll synthase (*CHLG*)*,* and chlorophyllide a oxygenase (*CAO*). Gene-specific primers are listed in Additional file 1: Table S1. *Actin7* was used as an internal Control. The relative level of gene expression was calculated using the 2^-∆∆CT^ method [39]. The gene transcription levels in Control plants were normalized as 1.

### Determination of GABA and Glu contents

GABA and Glu contents were measured by performing liquid chromatography-mass spectrometry (LC-MS, LC: AC, ExionLC; MS:Q-trap5500, AB Sciex). Leaves were washed with distilled water for 3 times before sampling. Fresh leaves (0.5 g) were homogenized with 1 ml 50% ethanol (include 0.1 M HCl), and centrifuged for 10 min at 12,000 *g* at 4 °C. The supernatant was collected for measurement. Methanol was added to bring the final volume of supernatant to 10 mL. The sample was filtered through a 0.22 μm filter before injecting into the LC-MS column (Intertisl OSD-4 C18; 250 mm × 3 mm, 5 μm). Sample injection volume was 20 μL, flow rate was 0.3 mL min^− 1^; mobile phase A was 0.1% methanoic acid, mobile phase B was methanol. Mass spectrometry conditions were as follows: electrospray ionization source (ESI), cation mode, spray voltage 4.5 kV, ion transfer capillary temperature was 275 °C, fragmentation and scanning of amino acids using data-dependent scanning. Detection (UV-detector) was performed at 330 nm. GABA retention time was 12 min. Data were quantified by comparing the peak surface areas with those obtained using pure GABA standards.

### Statistical analysis

All data were analyzed with SPSS 20 software (IBM) using Tukey’s multiple range test at a significance level of *P* < 0.05, unless stated otherwise; each reported data point represents the average of three biological replicates (*n* = 3) unless stated otherwise.

## Results

### Effects of exogenous GABA on muskmelon seedlings tolerance to salinity-alkalinity stress

**Fig. 1:**
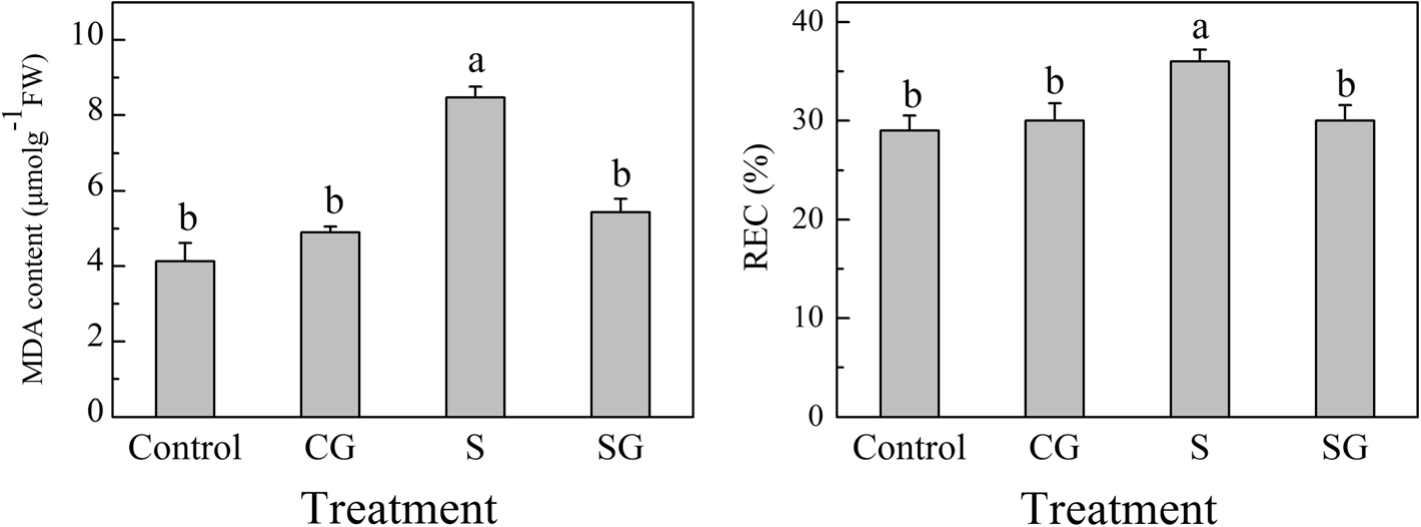
Effect of exogenous GABA on relative electrical conductivity (REC) and malondialdehyde (MDA) in muskmelon seedlings subjected to salinity-alkalinity stress at 3 d. H_2_O foliar prespraying for 8 h under normal nutrient solution cultivation conditions, (Control); 50 mM GABA foliar prespraying for 8 h under nutrient solution cultivation conditions, (CG); Normal nutrient solution containing 50 mM salinity-alkalinity and H_2_O foliar prespraying, (S); 50 mM GABA foliar prespraying for 8 h under salinity-alkalinity stress, (SG). Data were analyzed with SPSS 20 software (IBM) using Tukey’s multiple range test at a significance level of *P* < 0.05, and different letters above the bars indicate a significant difference. Data were expressed as the mean ± standard error of three independent biological replicates

Under normal growth conditions, pretreatment with exogenous GABA did not significantly affect fresh and dry weight, MDA content and REC except for area of leaf, compared with Control plants (*P* < 0.05) (Table 1 and Fig. 1).

**Table 1:** Effects of exogenous GABA on the growth of muskmelon seedlings exposed to salinity-alkalinity stress at 3 d

Treatment	Total fresh weight (g/plant)	Total dry weight (g/plant)	Area of leaf (cm^2^/plant)
Control	18.31 ± 1.42ab	1.08 ± 0.55ab	235.09 ± 15.6b
CG	25.08 ± 2.90a	1.39 ± 0.13a	289.16 ± 16.4a
S	9.54 ± 0.64c	0.72 ± 0.06b	158.76 ± 6.5c
SG	14.57 ± 0.76b	1.00 ± 0.06ab	173.95 ± 13.3bc

H_2_O foliar prespraying for 8 h under normal nutrient solution cultivation conditions, (Control); 50 mM GABA foliar prespraying for 8 h under nutrient solution cultivation conditions, (CG); Normal nutrient solution containing 50 mM salinity-alkalinity and H_2_O foliar prespraying, (S); 50 mM GABA foliar prespraying for 8 h under salinity-alkalinity stress, (SG). Data were analyzed with SPSS 20 software (IBM) using Tukey’s multiple range test at a significance level of *P* < 0.05, and different letters above the bars indicate a significant difference. Data were expressed as the mean ± standard error of three independent biological replicates

Salinity-alkalinity stress reduced the leaf area and total fresh and dry weights by 32.5, 49.9, and 7.4%, respectively, compared with Control plants (*P* < 0.05). By contrast, pretreatment with GABA mitigated the growth-inhibiting effects of salinity-alkalinity stress, and the total fresh weight significantly increased by 52.7% compared with that of plants subjected to salinity-alkalinity stress alone. Salinity-alkalinity stress increased REC and MDA content of muskmelon leaves (Fig. 1), whereas pretreatment with GABA significantly reduced stress-induced increases in REC and MDA content. We also investigated the effects of the GABA biosynthesis inhibitor 3-MP on the growth and membrane lipid peroxidation of melon grown under salinity-alkalinity stress (Additional file 2: Table S2, Additional file 3: Figure S1). Under stress conditions, 3-MP treatment further reduced plant growth indices, but no significant impact on REC, compared to S treatment. Whereas pretreatment with 3-MP plus GABA dramatically reduced the MDA content and REC, but increased the area of leaf compared with 3-MP + S treatment. These combined results indicated that exogenous GABA mitigated membrane lipid peroxidation in muskmelon and partially alleviated the salinity-alkalinity stress suppressed growth.

### Effects of exogenous GABA on antioxidant enzymes and nonenzymatic oxidants in muskmelon seedlings

**Fig. 2:**
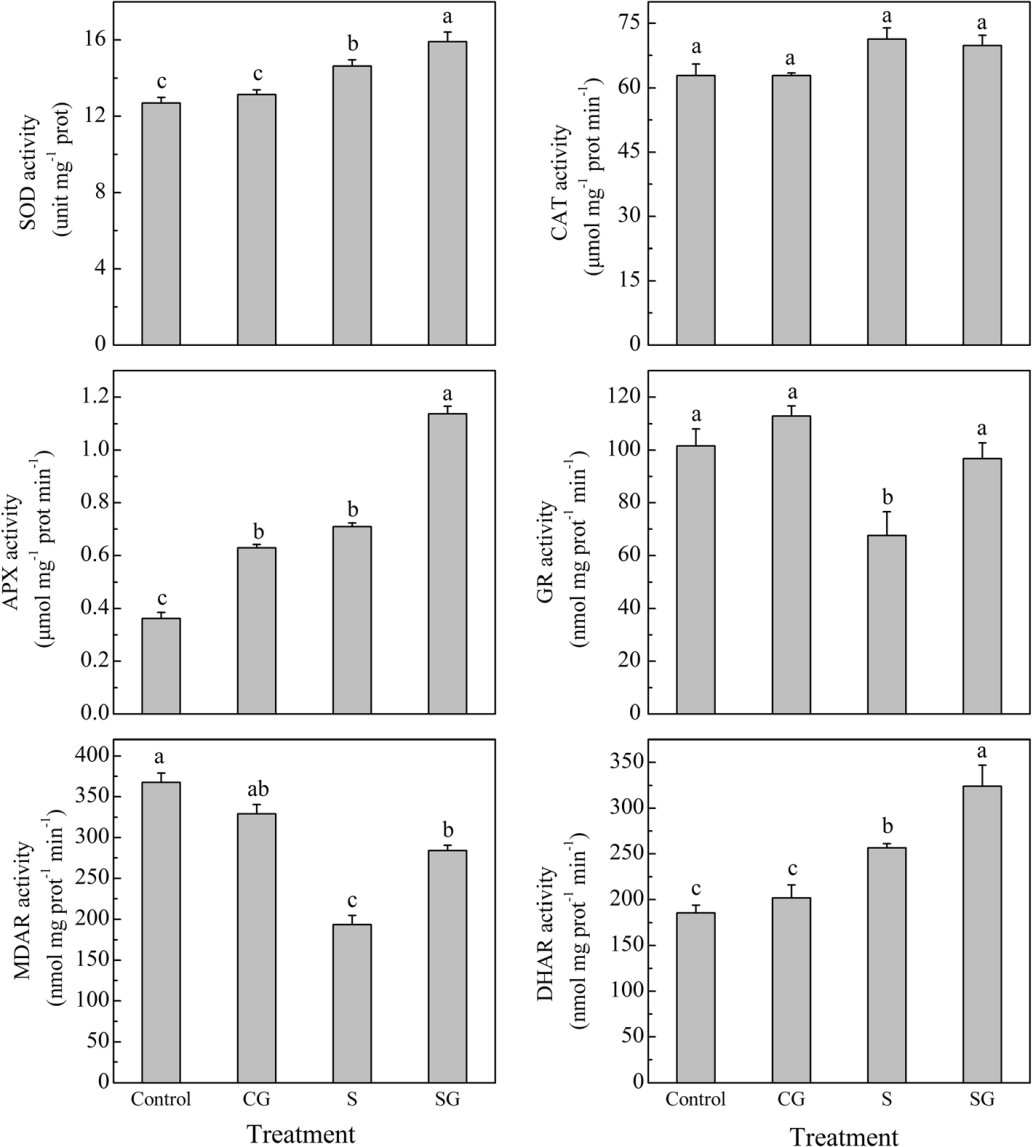
Effects of exogenous GABA on antioxidant enzymes in muskmelon seedlings subjected to salinity-alkalinity stress at 3 d. H_2_O foliar prespraying for 8 h under normal nutrient solution cultivation conditions, (Control); 50 mM GABA foliar prespraying for 8 h under nutrient solution cultivation conditions, (CG); Normal nutrient solution containing 50 mM salinity-alkalinity and H_2_O foliar prespraying, (S); 50 mM GABA foliar prespraying for 8 h under salinity-alkalinity stress, (SG). Data were analyzed with SPSS 20 software (IBM) using Tukey’s multiple range test at a significance level of *P* < 0.05, and different letters above the bars indicate a significant difference. Data were expressed as the mean ± standard error of three independent biological replicates

**Fig. 3:**
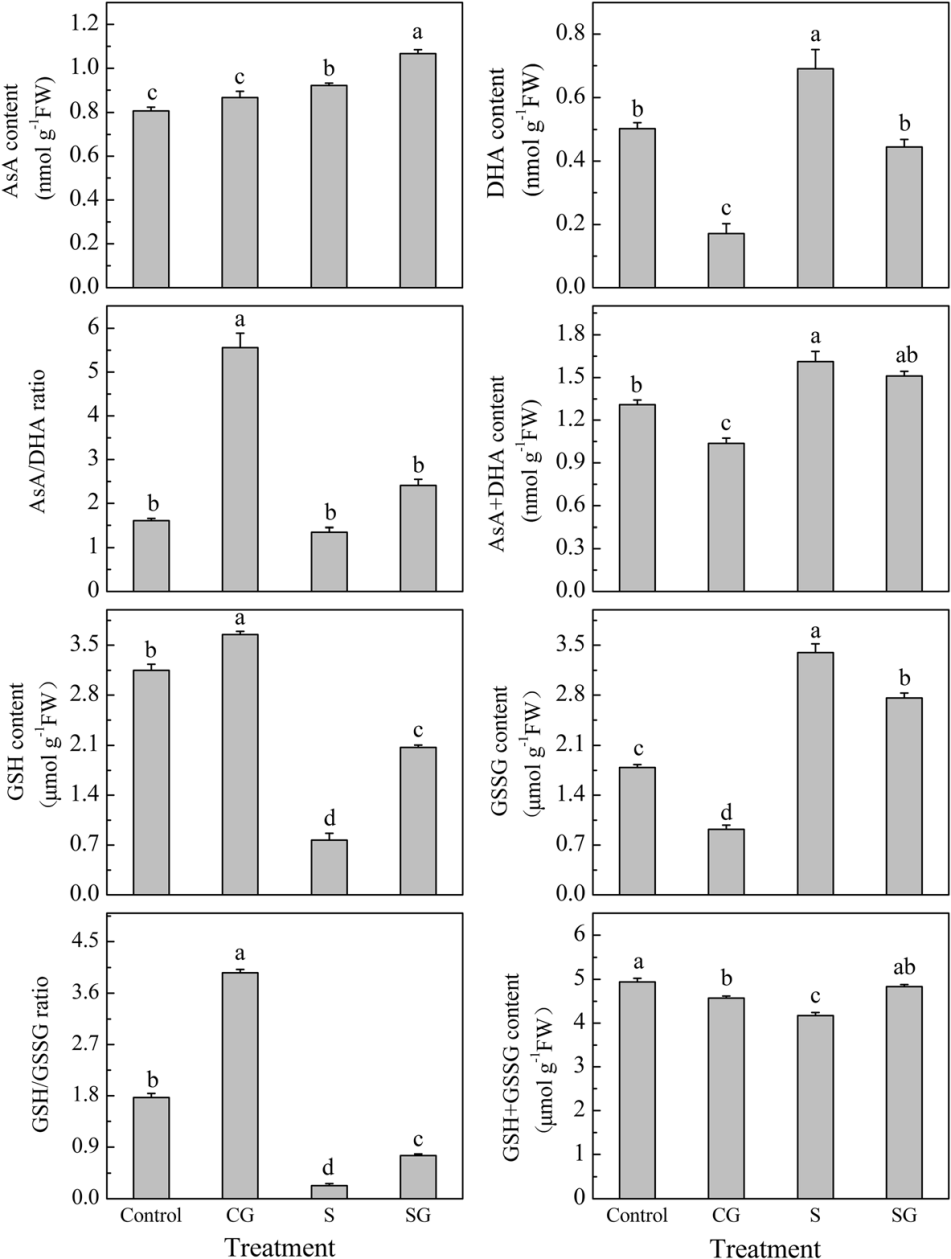
Effects of exogenous GABA on antioxidants in muskmelon seedlings subjected to salinity-alkalinity stress at 3 d. H_2_O foliar prespraying for 8 h under normal nutrient solution cultivation conditions, (Control); 50 mM GABA foliar prespraying for 8 h under nutrient solution cultivation conditions, (CG); Normal nutrient solution containing 50 mM salinity-alkalinity and H_2_O foliar prespraying, (S); 50 mM GABA foliar prespraying for 8 h under salinity-alkalinity stress, (SG). Data were analyzed with SPSS 20 software (IBM) using Tukey’s multiple range test at a significance level of *P* < 0.05, and different letters above the bars indicate a significant difference. Data were expressed as the mean ± standard error of three independent biological replicates

Antioxidant systems, including antioxidant enzymes and nonenzymatic oxidants, have crucial roles to mitigate cellular oxidation. As shown in Figs. 2 and 3, under normal conditions, GABA pretreatment has no significant effect on antioxidant enzymes, except for APX, which was dramatically elevated by 73.7% after GABA pretreatment (*P* < 0.05). Salinity-alkalinity stress significantly affected the activities of antioxidant enzymes: the activities of SOD, APX, and DHAR were increased by 15.2, 96.1, and 38.0%, respectively (*P* < 0.05); the activities of GR and MDAR were decreased by 33.5 and 47.4%, respectively (*P* < 0.05), compared with Control. GABA pretreatment under salinity-alkalinity stress conditions significantly increased the activities of SOD, APX, GR, DHAR, and MDAR by 8.7, 60.3, 43.4, 26.3, and 46.7%, respectively (*P* < 0.05), compared with the activities in plants subjected to salinity-alkalinity stress alone. GABA pretreatment under normal conditions enhanced the AsA/DHA ratio, GSH/GSSG ratio, and GSH contents, and reduced the DHA, AsA + DHA, GSSG, and GSSH+GSSG contents. Salinity-alkalinity stress enhanced the AsA, DHA, AsA + DHA, and GSSG contents, and reduced the contents of GSH, GSH + GSSG, and the GSH/GSSG ratio. Under salinity-alkalinity stress, pretreatment with GABA increased the contents of AsA, GSH, GSH + GSSG, and the AsA/DHA and GSH/GSSG ratios compared with salinity-alkalinity treatment alone. These combined results indicated that exogenous GABA regulated antioxidant systems to eliminate oxidative stress damage caused by salinity-alkalinity stress.

### Effects of H_2_O_2_, GSH, and AsA on GABA-induced tolerance to salinity-alkalinity stress in muskmelon seedlings

**Fig. 4:**
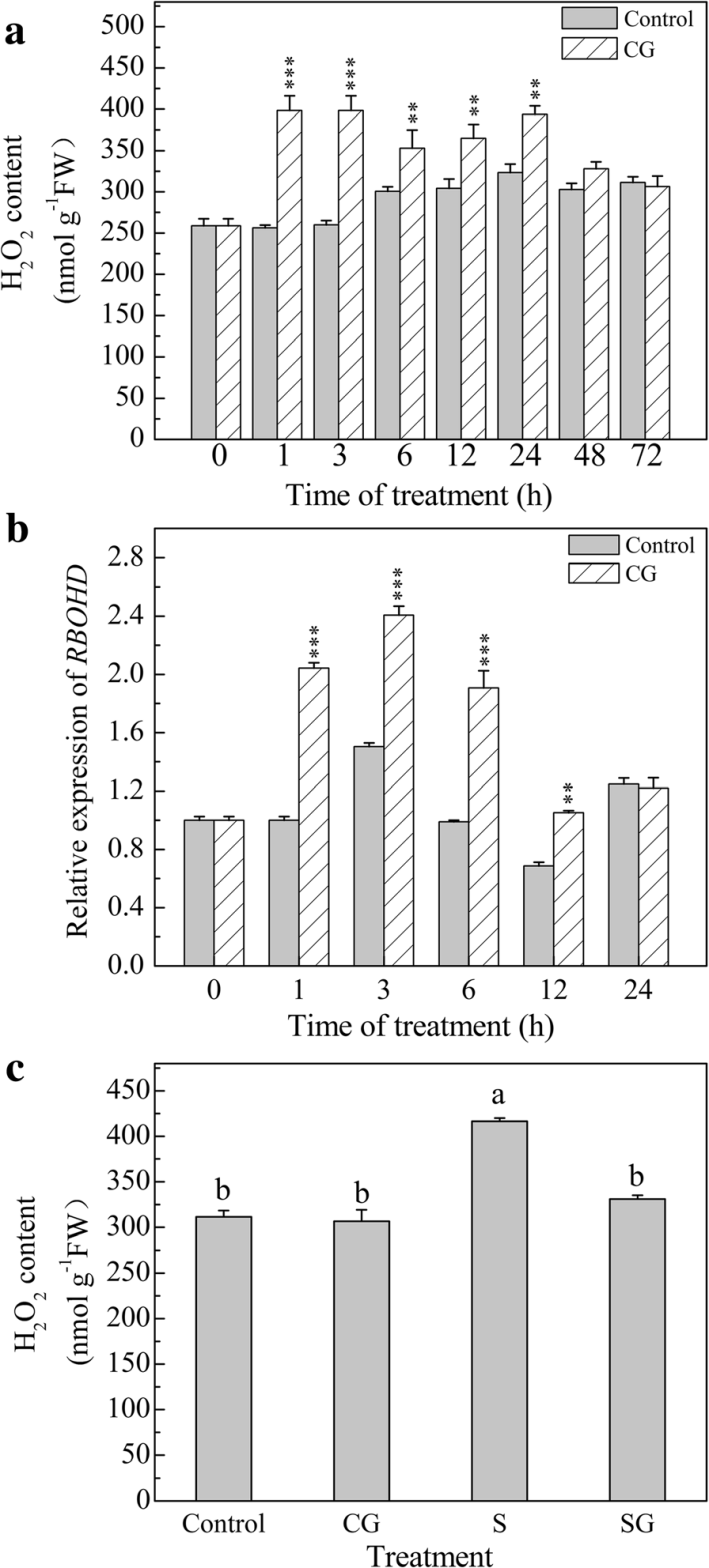
Effects of exogenous GABA on H_2_O_2_ levels in muskmelon seedlings. **a** Effects of exogenous GABA on H_2_O_2_ levels in muskmelon seedlings within 72 h. **b** Effects of exogenous GABA on the relative expression levels of *RBOHD* gene in muskmelon seedlings within 24 h. The gene transcription levels in Control plants at 0 h was normalized as 1. **c** Effects of exogenous GABA on H_2_O_2_ contents in muskmelon seedlings exposed to salinity-alkalinity stress at 3 d. H_2_O foliar prespraying for 8 h under normal nutrient solution cultivation conditions, (Control); 50 mM GABA foliar prespraying for 8 h under nutrient solution cultivation conditions, (CG); Normal nutrient solution containing 50 mM salinity-alkalinity and H_2_O foliar prespraying, (S); 50 mM GABA foliar prespraying for 8 h under salinity-alkalinity stress, (SG). Data were analyzed with SPSS 20 software (IBM) using Tukey’s multiple range test at a significance level of *P* < 0.05. Data are expressed as the mean ± standard error of three independent biological replicates. Significance is defined as follows: significant at *P < 0.05, **P < 0.01, ***P < 0.001

Under normal conditions, pretreatment with GABA induced a dramatically up-regulation of *RBOHD* gene expression from 1 h to 12 h and peaked at 3 h (Fig. 4b), while the GABA triggered significant accumulation of H_2_O_2_ from 1 h to 24 h, peaking at 3 h, compared with Control plants (*P <* 0.05, Fig. 4a). At 72 h, the H_2_O_2_ content in S treated plants was significant higher than Control plants, while pretreatment with GABA attenuated H_2_O_2_ accumulation compared with salinity-alkalinity stressed plants alone (Fig. 4c).

**Fig. 5:**
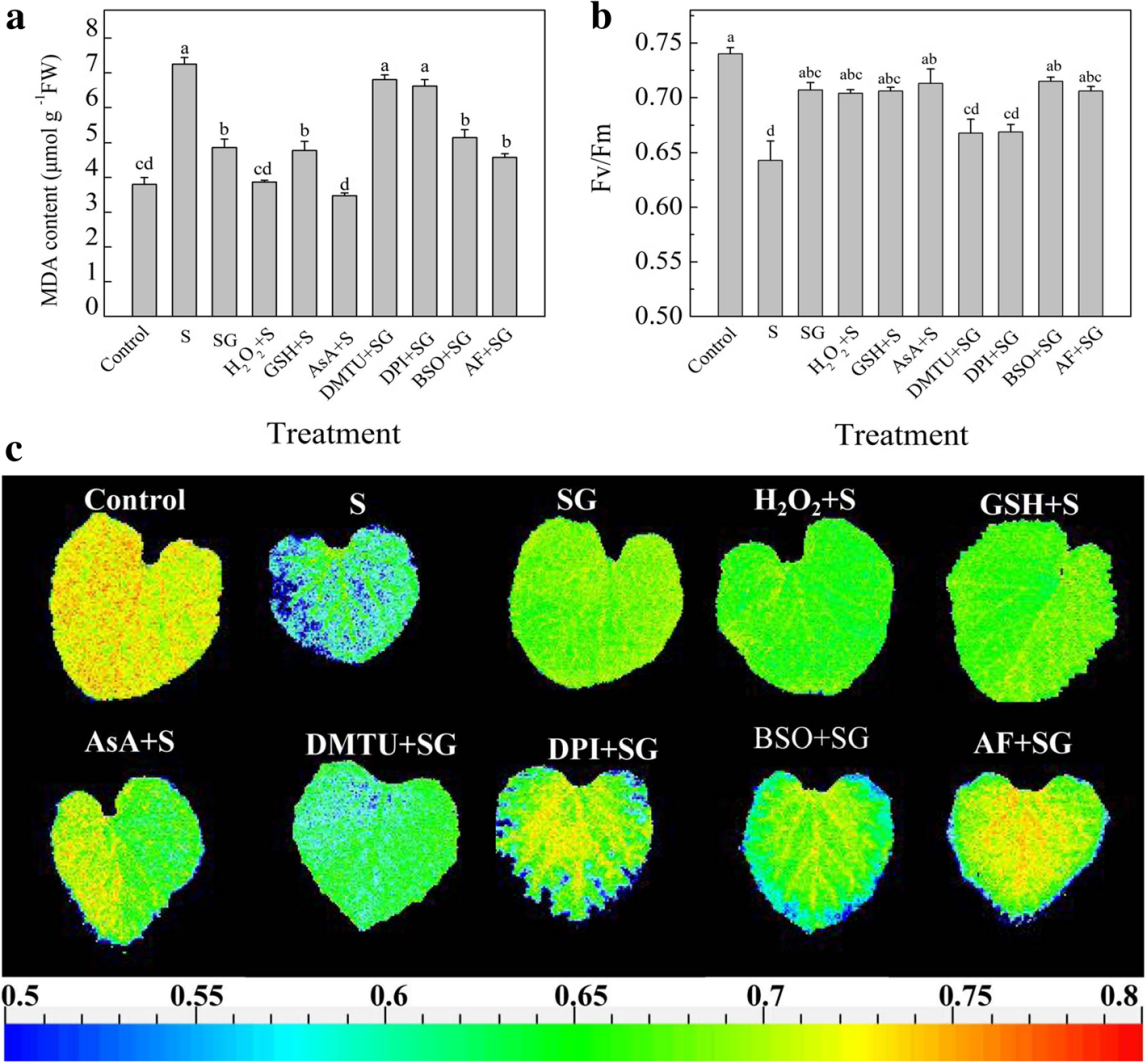
Evaluation of the effect of H_2_O_2_, GSH, and AsA in GABA-induced tolerance to salinity-alkalinity at 3 d in muskmelon seedlings. **a** The effect of H_2_O_2_, GSH, and AsA in GABA regulated MDA content under salinity-alkalinity stress. **b** The effect of H_2_O_2_, GSH, and AsA in GABA regulated Fv/Fm under salinity-alkalinity stress. **c** Images of the Fv/Fm, the false color code depicted at the bottom of the image ranges from 0.5 (blue) to 0.8 (red). H_2_O foliar prespraying for 8 h under normal nutrient solution cultivation conditions, (Control); Normal nutrient solution containing 50 mM salinity-alkalinity and H_2_O foliar prespraying, (S); 50 mM GABA foliar prespraying for 8 h under salinity-alkalinity stress, (SG); 5 mM H_2_O_2_ foliar prespraying for 8 h under salinity-alkalinity stress, (H_2_O_2_ + S); 5 mM GSH foliar prespraying for 8 h under salinity-alkalinity stress, (GSH + S); 1 mM AsA foliar prespraying for 8 h under salinity-alkalinity stress, (AsA + S); 5 mM DMTU foliar prespraying for 8 h, then spraying 50 mM GABA, after 8 h, treatment of salinity-alkalinity stress, (DMTU+SG); 100 μM DPI foliar prespraying for 8 h, then spraying 50 mM GABA, after 8 h, treatment of salinity-alkalinity stress, (DPI + SG); 1 mM BSO foliar prespraying for 8 h, then spraying 50 mM GABA, after 8 h, treatment of salinity-alkalinity stress, (BSO + SG); 1 mM AF foliar prespraying for 8 h, then spraying 50 mM GABA, after 8 h, treatment of salinity-alkalinity stress, (AF + SG). Data were analyzed with SPSS 20 software (IBM) using Tukey’s multiple range test at a significance level of *P* < 0.05, and different letters above the bars indicate a significant difference. Data were expressed as the mean ± standard error of three independent biological replicates

To further characterize the roles of H_2_O_2_, GSH, and AsA in GABA-induced tolerance to salinity-alkalinity stress in muskmelon, we pre-treated plants with DMTU (scavenges H_2_O_2_ and O_2_^−^), BSO (inhibits GSH biosynthesis), AF (inhibits AsA biosynthesis), and DPI (inhibits NADPH oxidases and oxidative burst) firstly before pretreatment with GABA, H_2_O_2_, GSH and AsA, and subsequently measured MDA content and F_v_/F_m_ (Fig. 5).

The results showed that pretreatment with GABA, H_2_O_2_, GSH, and AsA dramatically reduced the MDA contents by 32.8, 15.0, 33.7, and 42.9%, respectively, and increased F_v_/F_m_ by 9.9, 9.5, 9.8 and 10.9% respectively (*P* < 0.05), compared with salinity- alkalinity treatment alone. Pretreatment with DMTU or DPI significantly increased the MDA content and partially reduced the F_v_/F_m_, compared to the pretreatment with GABA under salinity-alkalinity stress conditions. By contrast, BSO or AF pre-treated plants plus GABA had no significant effects on the MDA content and F_v_/F_m_, compared to the GABA pre-treated plants.

### Effect of exogenous GABA on chlorophyll content in muskmelon seedlings subjected to salinity-alkalinity stress

**Fig. 6:**
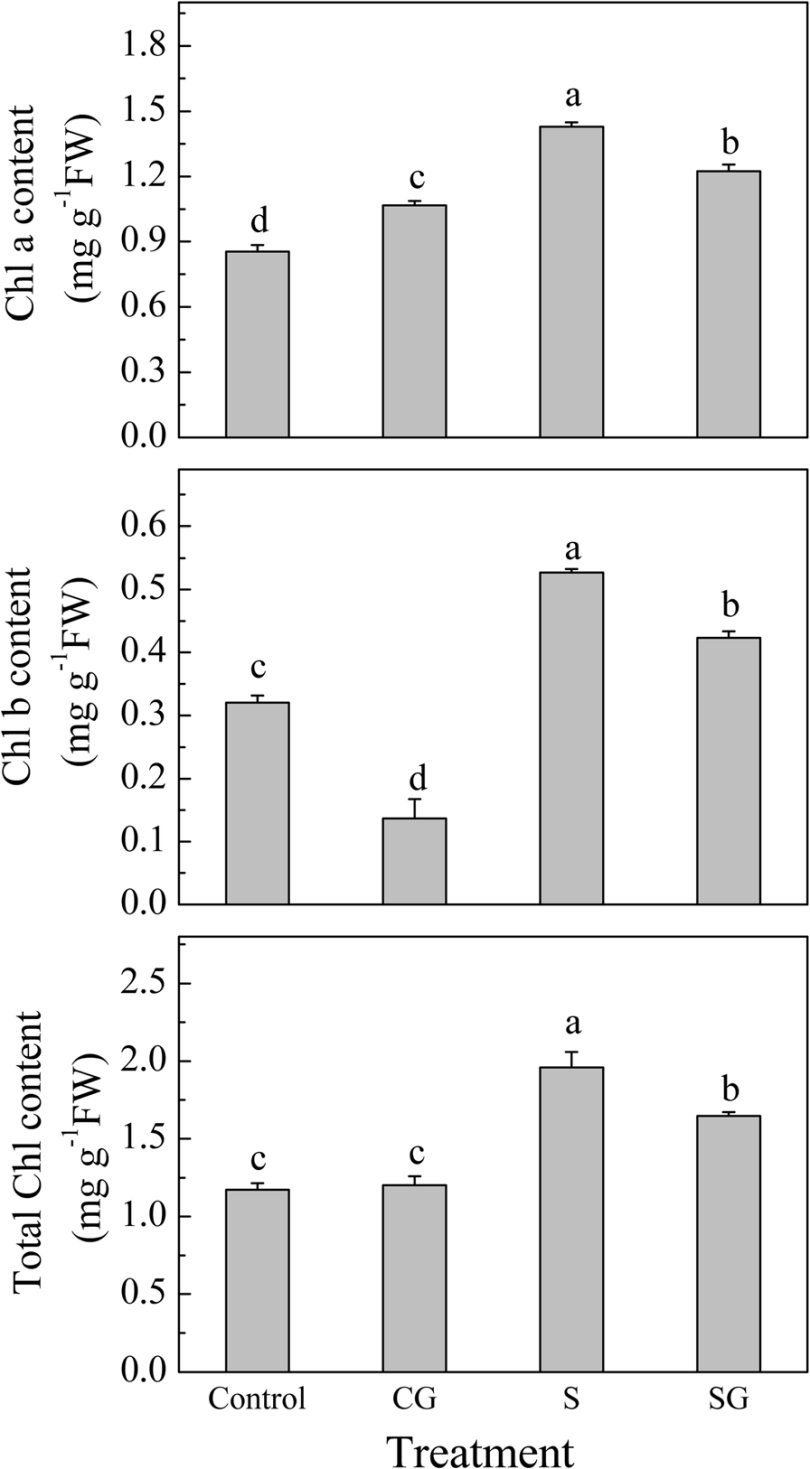
Effects of exogenous GABA on chlorophyll contents in muskmelon seedlings exposed to salinity-alkalinity stress at 3 d. H_2_O foliar prespraying for 8 h under normal nutrient solution cultivation conditions, (Control); 50 mM GABA foliar prespraying for 8 h under nutrient solution cultivation conditions, (CG); Normal nutrient solution containing 50 mM salinity-alkalinity and H_2_O foliar prespraying, (S); 50 mM GABA foliar prespraying for 8 h under salinity-alkalinity stress, (SG). Data were analyzed with SPSS 20 software (IBM) using Tukey’s multiple range test at a significance level of *P* < 0.05, and different letters above the bars indicate a significant difference. Data were expressed as the mean ± standard error of three independent biological replicates

Chlorophyll is essential for photosynthesis. Salinity-alkalinity stress negatively affects plant photosynthetic capacity [1, 3]. Therefore, we investigated Chl under salinity-alkalinity stress and GABA pretreatment. Under normal conditions, pretreatment with GABA enhanced the Chl a content and reduced the Chl b content, but did not significantly affect the total Chl content compared with Control plants (Fig. 6). Under salinity-alkalinity stress conditions, there was a significant increase in the contents of Chl a, Chl b, and total Chl compared with those of the Control plants (*P* < 0.05). Pretreatment with GABA before plants were subjected to salinity-alkalinity stress significantly reduced the contents of Chl a, Chl b, and total Chl compared with the stress treatment alone (*P* < 0.05).

### Effects of exogenous GABA on chlorophyll precursor content and gene expression in muskmelon seedlings subjected to salinity-alkalinity stress

**Fig. 7:**
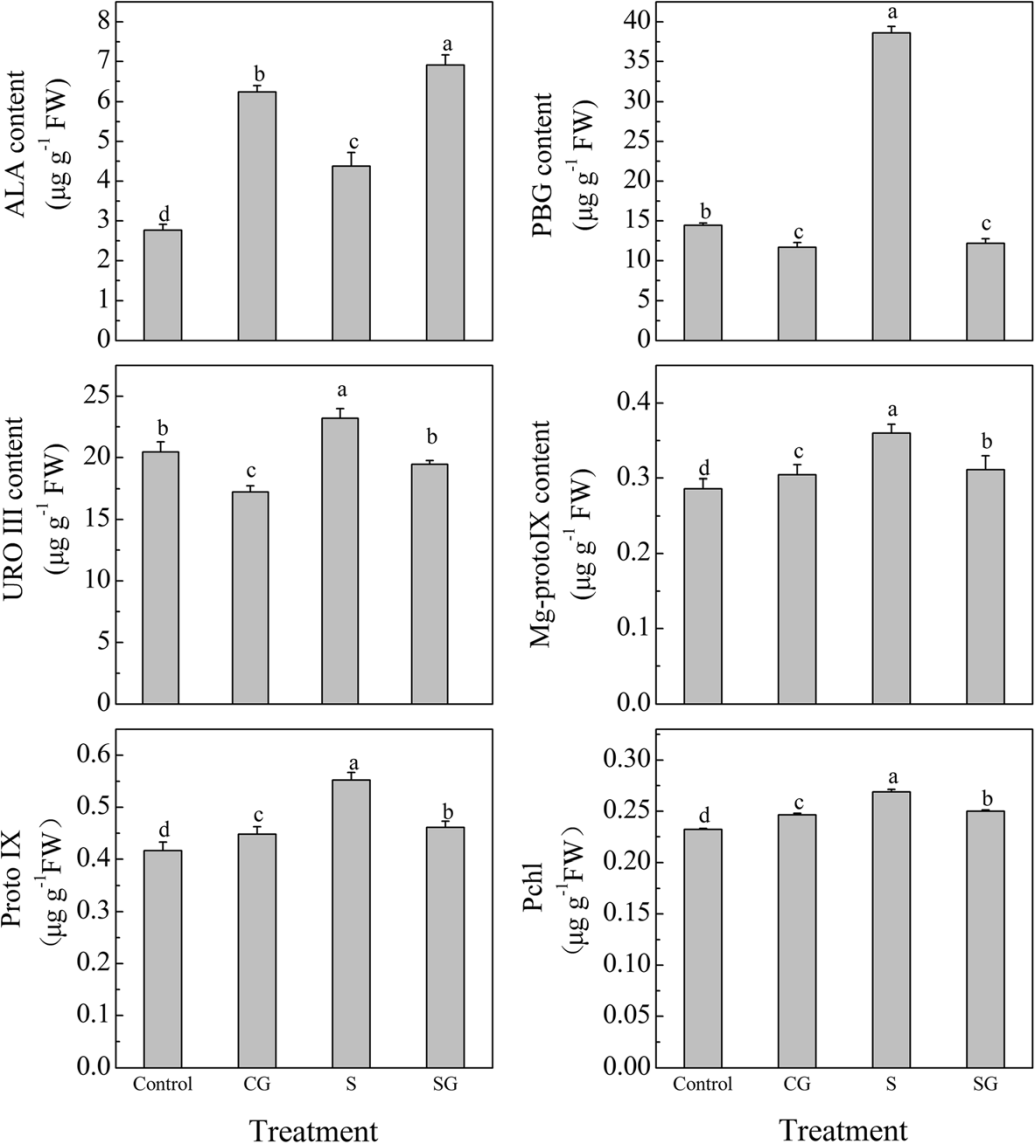
Effects of exogenous GABA on chlorophyll precursor contents in muskmelon seedlings exposed to salinity-alkalinity stress at 3 d. H_2_O foliar prespraying for 8 h under normal nutrient solution cultivation conditions, (Control); 50 mM GABA foliar prespraying for 8 h under nutrient solution cultivation conditions, (CG); Normal nutrient solution containing 50 mM salinity-alkalinity and H_2_O foliar prespraying, (S); 50 mM GABA foliar prespraying for 8 h under salinity-alkalinity stress, (SG). Data were analyzed with SPSS 20 software (IBM) using Tukey’s multiple range test at a significance level of *P* < 0.05, and different letters above the bars indicate a significant difference. Data were expressed as the mean ± standard error of three independent biological replicates

Under normal conditions, pretreatment with GABA significantly reduced the contents of PBG and URO III by 19.1 and 15.8%, respectively, and increased the contents of ALA, Mg-proto IX, Proto IX, and Pchl by 125.3, 6.3, 7.6, and 6.1%, respectively (*P* < 0.05, Fig. 7), compared with the Control. Under salinity-alkalinity stress, the contents of ALA, PBG, URO III, Mg-proto IX, Proto IX, and Pchl increased by 58.2, 167.5, 13.5, 25.6, 32.7, and 15.8%, respectively (*P* < 0.05), compared with the Control. Pretreatment with GABA under salinity-alkalinity stress significantly reduced the contents of the Chl precursors of PBG, URO III, Mg-proto IX, Proto IX, and Pchl by 68.4, 16.2, 13.5, 16.6, and 7.1%, respectively (*P* < 0.05), and increased the ALA content by 57.8% (*P* < 0.05), compared with the stress treatment alone (Fig. 7).

**Fig. 8:**
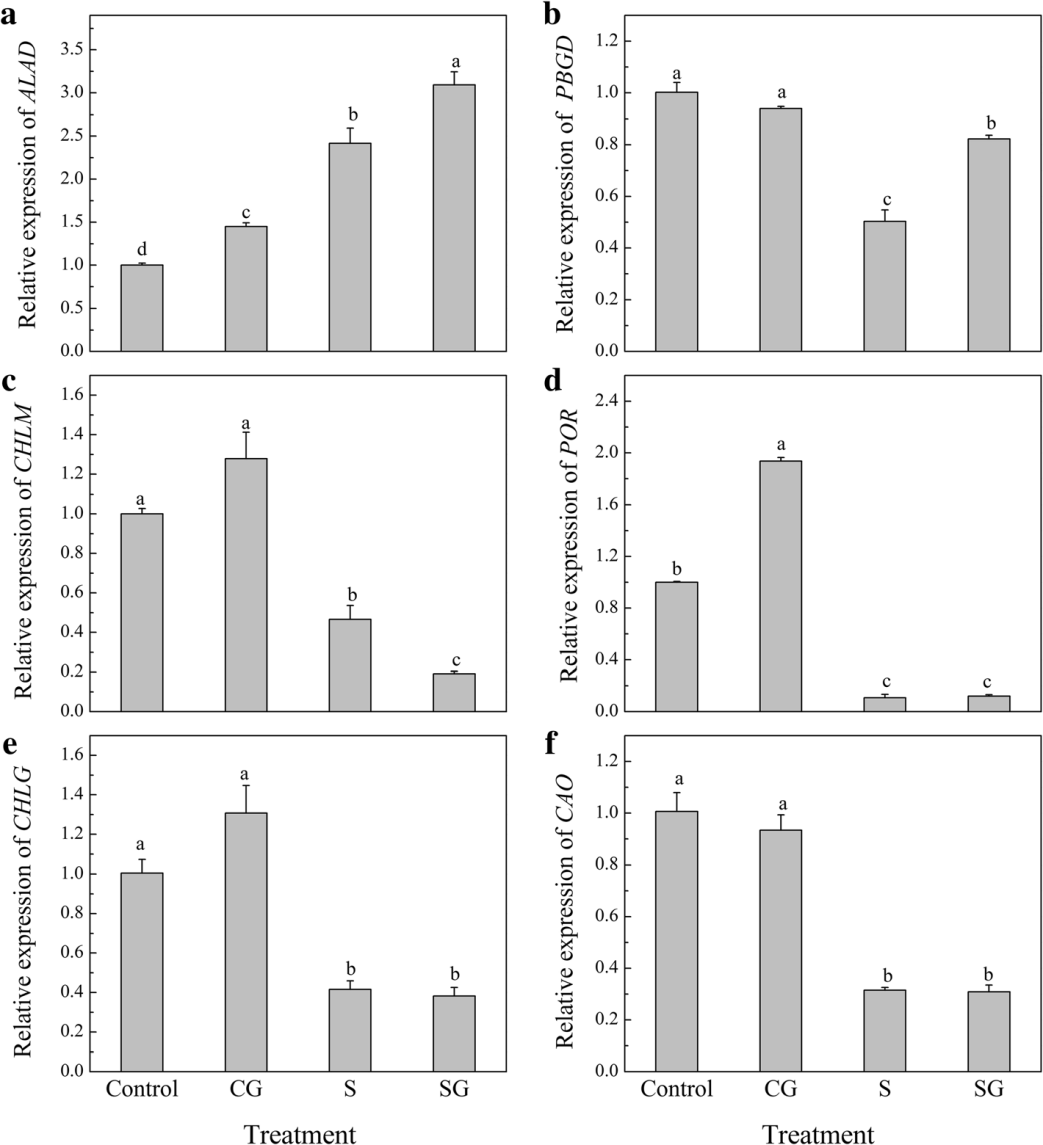
Effects of exogenous GABA on the relative expression levels of chlorophyll biosynthesis enzyme genes at 48 h. **a**-**f** represent the genes expression of *ALAD*, *PBGD*, *CHLM*, *POR*, *CHLG* and *CAO*, respectively. H_2_O foliar prespraying for 8 h under normal nutrient solution cultivation conditions, (Control); 50 mM GABA foliar prespraying for 8 h under nutrient solution cultivation conditions, (CG); Normal nutrient solution containing 50 mM salinity-alkalinity and H_2_O foliar prespraying, (S); 50 mM GABA foliar prespraying for 8 h under salinity-alkalinity stress, (SG). The gene transcription levels in Control plants was normalized as 1. Data were analyzed with SPSS 20 software (IBM) using Tukey’s multiple range test at a significance level of *P* < 0.05, and different letters above the bars indicate a significant difference. Data were expressed as the mean ± standard error of three independent biological replicates

Under normal condition, pre-treatd GABA significantly up-regulated the expression of *ALAD* and *POR* (*P* < 0.05, Fig. 8), compared to Control plants. Salinity-alkalinity stress increased the expression of *ALAD* (*P* < 0.05), but decreased other genes expression, compared to Control plants. GABA pre-treated plants plus salinity-alkalinity stress dramatically elevated the expression of *ALAD* and *PBGD*, and significantly declined the *CHLM* expression (*P* < 0.05).

### Effect of exogenous GABA on the contents of endogenous GABA and Glu in muskmelon seedlings under salinity-alkalinity stress

**Fig. 9:**
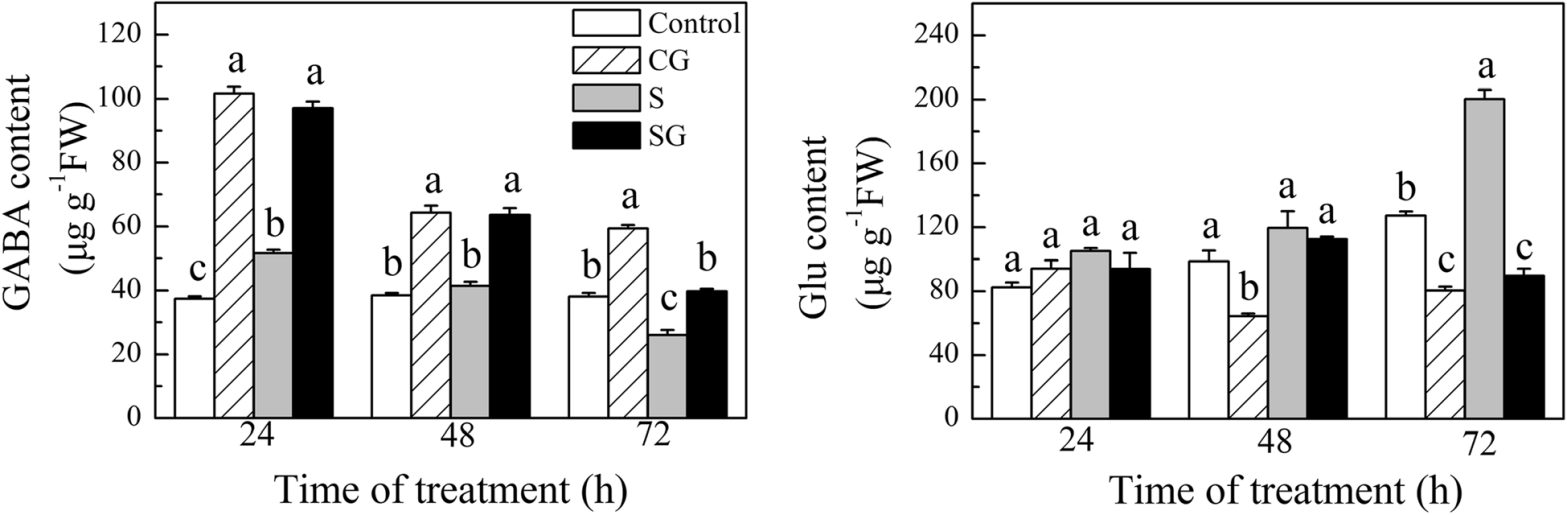
Effects of exogenous GABA on endogenous GABA and glutamate (Glu) contents in muskmelon seedlings exposed to salinity-alkalinity stress within 3 d. H_2_O foliar prespraying for 8 h under normal nutrient solution cultivation conditions, (Control); 50 mM GABA foliar prespraying for 8 h under nutrient solution cultivation conditions, (CG); Normal nutrient solution containing 50 mM salinity-alkalinity and H_2_O foliar prespraying, (S); 50 mM GABA foliar prespraying for 8 h under salinity-alkalinity stress, (SG). Data were analyzed with SPSS 20 software (IBM) using Tukey’s multiple range test at a significance level of *P* < 0.05, and different letters above the bars indicate a significant difference. Data were expressed as the mean ± standard error of three independent biological replicates

Chl biosynthesis begins with the synthesis of ALA which synthesized from glutamate (Glu) [40, 41]. Glu also is the precursor for GABA biosynthesis. Therefore, we measured the endogenous GABA and Glu contents in plants subjected to salinity-alkalinity stress for 24, 48, and 72 h (Fig. 9). Under normal conditions, pretreatment with exogenous GABA significantly increased the content of endogenous GABA (*P* < 0.05), which then declined in a time-dependent manner. This treatment dramatically reduced the Glu content compared with the Control at 48 h and 72 h (*P* < 0.05). The observed variation in endogenous GABA content was similar under salinity-alkalinity stress treatment and GABA treatment; at 72 h, the endogenous GABA content of S treatment was lower than that in the Control, while SG treatment increased the endogenous GABA content, compared to S treated plants. By contrast, under normal condition, exogenous GABA decreased the Glu content at 48 h and 72 h, while it was no significant difference at 24 h (Fig. 9). The Glu content in S- treated plant was higher than Control, while pretreatment with exogenous GABA under salinity-alkalinity stress significantly decreased the Glu content compared to S treated plant alone, at 72 h (*P* < 0.05, Fig. 9). In all, exogenous GABA promotes the accumulation of endogenous GABA and inhibits the accumulation of Glu.

## Discussion

### Exogenous GABA improved salinity-alkalinity stress tolerance by regulating the antioxidant system in muskmelon seedlings

High salinity-alkalinity conditions induce osmotic and oxidative stresses that perturb plant metabolism and limit plant growth and development [1, 42]. Previous study indicated that stress triggered excessive ROS production is tightly linked to cell membrane damage and electrolyte leakage [43]. In the present study, increased growth parameters such as fresh weight, dry weight, and leaf area indicated that foliar pretreatment with GABA effectively mitigated the growth limitation and membrane lipid oxidation induced by salinity-alkalinity stress (Table 1; Fig. 1). A previous study indicated that pretreatment with GABA mitigated stress-induced reduction of net photosynthesis and recovered the stress-induced damage to chloroplast structure [1]. Inhibition of endogenous GABA partially aggravated the damage caused by salinity-alkalinity stress, while fed with exogenous GABA mitigated the damage (Additional file 2: Table S2, Additional file 3: Figure S1). This said that endogenous GABA, as a signal molecular, may also play part roles on plant stress tolerance [16, 44]. Reduced growth and stress tolerance in endogenous GABA inhibited plants could be reversed and improved by the addition of exogenous GABA. These combined results demonstrated that exogenous GABA mitigated salinity-alkalinity stress in muskmelon seedlings. However, there was no strong evidence showing that GABA directly scavenged ROS to relieve the stress. Therefore, GABA-enhanced stress tolerance may be mediated via the antioxidant system.

Our results also indicated that salinity-alkalinity stress differentially affected the activities of antioxidant enzymes and the contents of nonenzymatic antioxidants. SOD is a key enzyme that converts O_2_^−^ into H_2_O_2_. CAT, APX and GPOX have essential roles in converting H_2_O_2_ to water and oxygen [45]. AsA-GSH is recognized as a key player in H_2_O_2_ metabolism in plants [46]. APX, GR, DHAR, and MDAR are involved in the AsA-GSH cycle and important for the regeneration of AsA and GSH. When H_2_O_2_ is reduced by APX, the AsA electron donor is oxidized to MDHA. AsA can be regenerated by MDHA and DHA via MDAR and DHAR enzymes, respectively. GSH is an important component of the AsA-GSH metabolic cycle, and is directly or indirectly involved in clearing ROS. GSH is a basic substrate in DHAR-mediated catalytic regeneration of AsA [46]. GR activity affects the level of GSH; therefore, GR is an important enzyme in plant stress responses [47]. GABA enhanced SOD activity, thereby eliminating the ROS generated by salinity-alkalinity stress, and promoting the conversion of O_2_^−^ to H_2_O_2_ (Fig. 2). In the present study, GABA increased the levels of AsA and GSH, and the activities of APX, GR, DHAR, and MDAR involved in the AsA-GSH cycle, but did not significantly affect CAT activity (Figs. 2 and 3). This result indicated that the AsA-GSH cycle, but not CAT, plays a major role in GABA regulated reduction of H_2_O_2_. Salinity-alkalinity stress reduced MDAR activity but increased DHAR activity, while pretreatment with GABA elevated the MDAR and DHAR activities (Fig. 2). These results demonstrated that MDAR was suppressed, and DHAR was the main enzyme for regenerating AsA in S treated plants. And AsA regeneration in GABA treated plants was due to the DHAR and MDAR under salinity-alkalinity stress. This said that GABA eliminated the S suppressed MDAR activity. Exogenous pretreatment with GABA under salinity-alkalinity stress increased GR activity and GSH content (Figs. 2 and 3), indicating that high GR activity is the primary mechanism for maintaining the GSH level in plant cells [48].

### H_2_O_2_ has an important role in GABA-induced salinity-alkalinity stress tolerance

Many studies demonstrated that ROS is an important signal molecule that regulates plant growth, metabolism and stress response [16, 23, 24]. Additionally, Bao et al. [16] suggested that GABA itself may not directly correlate with salt tolerance. In present study, GABA treatment induced *RBOHD* gene expression and H_2_O_2_ accumulation under normal conditions (Fig. 4a and b), whereas GABA reduced the H_2_O_2_ content under 3 d of salinity-alkalinity stress (Fig. 4c). Under salinity-alkalinity stress, pretreatment with GABA, H_2_O_2_, GSH, and AsA enhanced the salinity-alkalinity stress tolerance; but inhibiting the production of endogenous H_2_O_2_ attenuated theses positive effects, while inhibiting the production of endogenous GSH and AsA did not significantly affect GABA roles on MDA content and Fv/Fm (Fig. 5). Taken together, we speculated that exogenous GABA elevated H_2_O_2_ level under normal condition via up-regulating the *RBOHD* genes expression which encoded the NADPH oxidases [24]. This kind of H_2_O_2_ in the apoplast and may act as a signaling molecule responded to the stress and then triggered the antioxidant system to cope with salinity-alkalinity stress caused excessive ROS accumulation and membrane lipid damage [49], whereas GSH and AsA may be antioxidants mediating the ROS balance.

### Exogenous GABA regulates chlorophyll biosynthesis under salinity-alkalinity stress

Chl is essential for photosynthesis. Reduced photosynthetic capacity is the primary cause for plant growth inhibition when subjected to salinity-alkalinity stress. Previous studies showed that salinity-alkalinity stress negatively affected photosynthesis in developed leaves [1, 50]. Chl biosynthesis in plants proceeds through a series of reactions, and Chl biosynthesis is affected if any of these steps are disrupted [51, 52].

Many studies have shown that salinity stress disturbed Chl metabolism [3, 53, 54]. Under normal condition, GABA pretreatment significantly increased the Chl a content and decreased Chl b content compared to Control plants (Fig. 6), this may be due to the higher Chl precursor contents in GABA treated plants (Fig. 7). The high Chl a may be more conducive for plant light energy conversion and improvement of photosynthesis efficiency. Under normal condition, prespraying GABA slows the transformation from Chl a to Chl b, which resulted in higher Chl a and lower Chl b contents. We demonstrated that Chl contents were sharply enhanced after 3 days of salinity-alkalinity stress (Fig. 6), which may be also due to the elevated Chl precursor contents (Fig. 7). Glu is the common precursor for ALA and GABA [55]. Our study found that salinity-alkalinity stress enhanced the Glu content, promoted Glu conversion into ALA, and inhibited the conversion of Glu into GABA; this may be the reason why ALA content increased and GABA content declined over time (Figs. 7 and 9). Other studies have reported similar effects of salinity or lower temperature on increased Glu accumulation [56]. The exogenous GABA may be absorbed directly by plants [57–59], which then engaged in downstream metabolisms and were converted into other substances [16]. This may be the reason why endogenous GABA levels in GABA-treated plants was higher than untreated plants, although it appeared the highest value at the beginning, and gradually decreasing with the extension of time. Excess accumulation of amino acids under stress conditions might indicate cell damage in some species [60]. Wang et al. [53] suggested that UV-B disrupts Chl synthesis at the point of ALA conversion to PBG. Li et al. [3] suggested that salinity-alkalinity stress blocks the conversion of URO III into Proto IX. This difference may be crop or cultivar-specific [61]. In this study, compared with Control, salinity-alkalinity stress triggered a accumulation of Chl and its precursor contents, while GABA pretreatment reversed these trends under salinity-alkalinity stress (Fig. 7). However, these genes expression show a inconsistent trends with the Chl precursor contents (Fig. 8). These results said that salinity-alkalinity stress did not inhibit the Chl biosynthesis which was regulated at a level other than transcription. In addition, the over-accumulation of Chl and its precursors under stress may trigger photooxidation injury [62–64], which ultimately caused membrane lipid damage (Fig. 1) and affects the plant growth (Table. 1). Under salinity-alkalinity stress, pretreatment with GABA reduced the over-accumulation of Chl a and its precursors, which then reduced the Chl b content, may be crucial for avoiding the photooxidation injury. Our previous research also showed that GABA could maintain chloroplast structure and membrane integrity [1]. In this study, GABA induced initial H_2_O_2_ signal increased plant stress response, which then improved the antioxidant ability and alleviated the membrane lipid peroxidation injury, which was also crucial for coordinated chlorophyll synthesis.

## Conclusions

Exogenous GABA has a positive effect on mitigating salinity-alkalinity stress by regulating the antioxidant system and Chl biosynthesis. GABA induced H_2_O_2_ may function as a signal molecule, whereas AsA and GSH function as antioxidants, involved in GABA-induced antioxidation to alleviate oxidative stress resistance. These factors maintain membrane integrity which was essential for the ordered chlorophyll biosynthesis. Pretreatment with GABA mitigated salinity-alkalinity stress caused excessive accumulation of Chl and its precursors, to avoid photooxidation injury.

## Additional files

Additional file 1: **Table S1.** Gene-specific primers designed for qRT-PCR. (DOCX 14 kb)

Additional file 2: **Table S2.** Plant growth with treatment of exogenous GABA or GABA biosynthesis inhibitor in muskmelon seedlings subjected to salinity-alkalinity stress at 3 d. Normal nutrient solution containing 50 mM salinity-alkalinity and H_2_O foliar prespraying, (S); 0.1 mM GABA biosynthesis inhibitor 3-mercaptopropionic (3-MP) foliar prespraying for 12 h under salinity-alkalinity stress, (3-MP + S); 0.1 mM 3-MP foliar prespraying for 12 h, then spraying 50 mM GABA, after 8 h, treatment of salinity-alkalinity stress, (3-MP + SG). Data were analyzed with SPSS 20 software (IBM) using Tukey’s multiple range test at a significance level of *P* < 0.05, and different letters above the bars indicate a significant difference. Data were expressed as the mean ± standard error of three independent biological replicates. (DOCX 13 kb)

Additional file 3: **Figure S1.** Relative electrical conductivity (REC) and malondialdehyde (MDA) with treatment of exogenous GABA or GABA biosynthesis inhibitor in muskmelon seedlings subjected to salinity-alkalinity stress at 3 d. Normal nutrient solution containing 50 mM salinity-alkalinity and H_2_O foliar prespraying, (S); 0.1 mM GABA biosynthesis inhibitor 3-mercaptopropionic (3-MP) foliar prespraying for 12 h under salinity-alkalinity stress, (3-MP + S); 0.1 mM 3-MP foliar prespraying for 12 h, then spraying 50 mM GABA, after 8 h, treatment of salinity-alkalinity stress, (3-MP + SG). Data were analyzed with SPSS 20 software (IBM) using Tukey’s multiple range test at a significance level of *P* < 0.05, and different letters above the bars indicate a significant difference. Data were expressed as the mean ± standard error of three independent biological replicates. (JPG 1382 kb)

## Abbreviations

3-MP: 3-mercaptopropionic
AF: Acriflavine
APX: Ascorbate peroxidase
AsA: Reduced ascorbate
BSO: Buthionine sulfoximine
CAT: Catalase
Chl: Chlorophyll
DHA: Dehydroascorbate
DHAR: Dehydroascorbate reductase
DMTU: Dimethylthiourea
DPI: Diphenyleneiodonium
Fv/Fm: Maximal quantum yield of PSII photochemistry
GR: Glutathione reductase
GSH: Reduced glutathione
GSSG: Oxidized glutathione
H_2_O_2_: Hydrogen peroxide
MDAR: Monodehydroascorbate reductase
Mg–proto IX: Mg–protoporphyrin IX
O_2_^−^: Superoxide anions
PBG: Porphobilinogen
Pchl: Protochlorophyll
Proto IX: Protoporphyrin IX
PSII: Photosystem I
ROS: Reactive oxygen species
SOD: Superoxide dismutase
URO III: Uroorphyrinogen III
