# Supplementary material for: Exogenous GABA enhances muskmelon tolerance to salinity-alkalinity stress by regulating redox balance and chlorophyll biosynthesis
Source: BMC Plant Biol. 2019 Feb 1;19:48. doi: 10.1186/s12870-019-1660-y (PMC6359809; doi:10.1186/s12870-019-1660-y)
Supplement: Supplementary file 1 — Table S1. Gene-specific primers designed for qRT-PCR. (DOCX 14 kb) [file 12870_2019_1660_MOESM1_ESM.docx]

Table S1. Gene-specific primers designed for qRT-PCR.

| **Gene name** | **Accession numbers** | **Forward primer** | **Reverse primer** |
| --- | --- | --- | --- |
| *Actin7* | XM008442791 | ATTCTTGCATCTCTAAGTACCTTCC | CCAACTAAAGGGAAATAACTCACC |
| *ALAD* | XM010326888 | ATGCCTGGATGTTATAGACTTGC | GATCAGGGTATTTGTCCTTGAGC |
| *PBGD* | XM008463618 | CCCTCAAAGTGCTGGAGAATT | ATTATGTCATCGTCACTTCGACAG |
| *POR* | XM004251804 | GGGGAAATCTAGTGTATGCTTGAA | CAGTTGTCTGAGCCCTTATTGC |
| *CHLM* | XM004235797 | CTGTAGCCACTGAACTTCCCATC | CATCACATTCTCCACCGTCTTG |
| *CHLG* | XM004246270 | GCGTTGACACTAAATACACCTCC | AGAAACAGAAACTGGGGATAGAGA |
| *CAO* | XM004250263 | ATCCTCTAGCCCGTGACTATAAAT | GTGGCAACTCGTGCTTCAGATA |
| *RBOHD* | XM008466943 | CAGTAGCGTCAGAAGGGGTG | CTATGGACGGCGACAGAGTC |
