# Supplementary material for: Exogenous GABA enhances muskmelon tolerance to salinity-alkalinity stress by regulating redox balance and chlorophyll biosynthesis
Source: BMC Plant Biol. 2019 Feb 1;19:48. doi: 10.1186/s12870-019-1660-y (PMC6359809; doi:10.1186/s12870-019-1660-y)
Supplement: Supplementary file 2 — Table S2. Plant growth with treatment of exogenous GABA or GABA biosynthesis inhibitor in muskmelon seedlings subjected to salinity-alkalinity stress at 3 d. Normal nutrient solution containing 50 mM salinity-alkalinity and H2O foliar prespraying, (S); 0.1 mM GABA biosynthesis inhibitor 3-mercaptopropionic (3-MP) foliar prespraying for 12 h under salinity-alkalinity stress, (3-MP + S); 0.1 mM 3-MP foliar prespraying for 12 h, then spraying 50 mM GABA, after 8 h, treatment of salinity-alkalinity stress, (3-MP + SG). Data were analyzed with SPSS 20 software (IBM) using Tukey’s multiple range test at a significance level of P < 0.05, and different letters above the bars indicate a significant difference. Data were expressed as the mean ± standard error of three independent biological replicates. (DOCX 13 kb) [file 12870_2019_1660_MOESM2_ESM.docx]

| Treatment | Total fresh weight (g/plant) | Total dry weight (g/plant) | Area of leaf  (cm^2^/plant) |
| --- | --- | --- | --- |
| S | 9.54±0.64b | 0.72±0.06a | 158.76±6.5ab |
| 3-MP+S | 8.5±0.90b | 0.73±0.08a | 129.33±15.3b |
| 3-MP+SG | 9.3±1.48b | 0.80±0.11a | 167.49±16.2a |

Table S2
