# Supplementary figures and images for: Exogenous GABA enhances muskmelon tolerance to salinity-alkalinity stress by regulating redox balance and chlorophyll biosynthesis
Source: BMC Plant Biol. 2019 Feb 1;19:48. doi: 10.1186/s12870-019-1660-y (PMC6359809; doi:10.1186/s12870-019-1660-y)

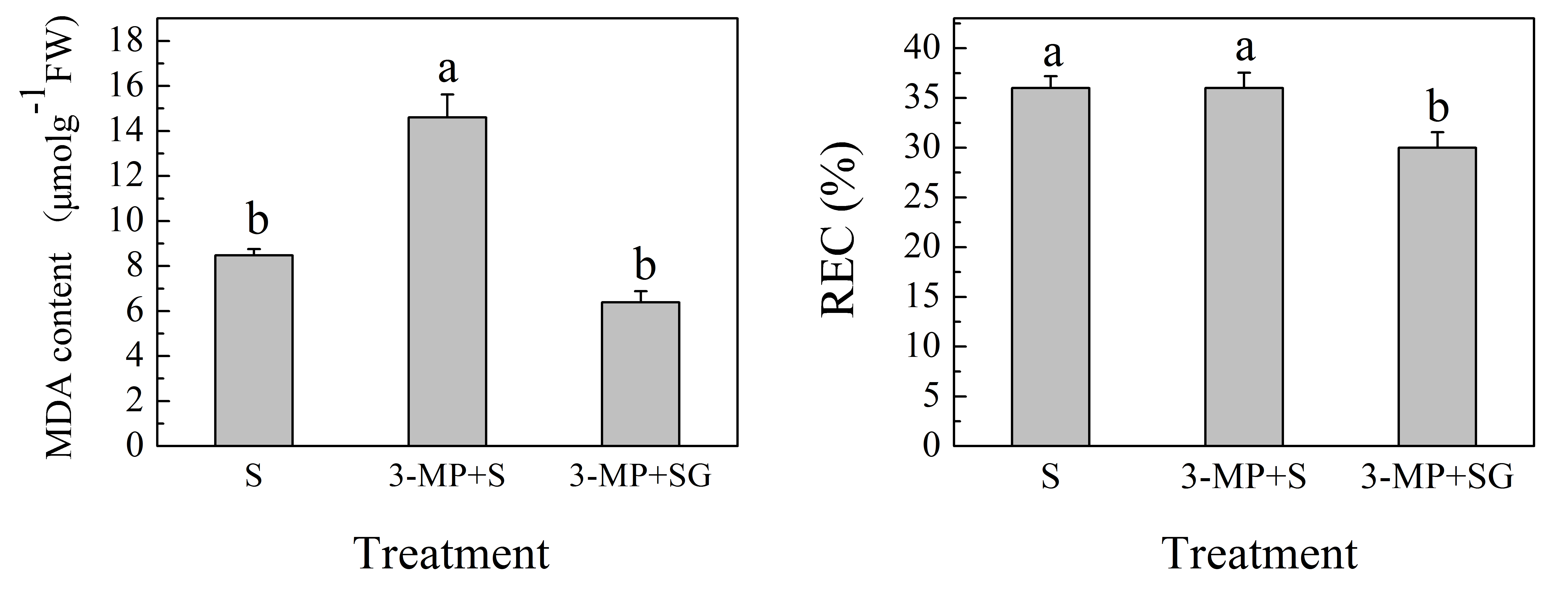

Supplement: Supplementary file 3 — Figure S1. Relative electrical conductivity (REC) and malondialdehyde (MDA) with treatment of exogenous GABA or GABA biosynthesis inhibitor in muskmelon seedlings subjected to salinity-alkalinity stress at 3 d. Normal nutrient solution containing 50 mM salinity-alkalinity and H2O foliar prespraying, (S); 0.1 mM GABA biosynthesis inhibitor 3-mercaptopropionic (3-MP) foliar prespraying for 12 h under salinity-alkalinity stress, (3-MP + S); 0.1 mM 3-MP foliar prespraying for 12 h, then spraying 50 mM GABA, after 8 h, treatment of salinity-alkalinity stress, (3-MP + SG). Data were analyzed with SPSS 20 software (IBM) using Tukey’s multiple range test at a significance level of P < 0.05, and different letters above the bars indicate a significant difference. Data were expressed as the mean ± standard error of three independent biological replicates. (JPG 1382 kb) [file 12870_2019_1660_MOESM3_ESM.jpg]
